# Reply: Estimation of lead-time and overdiagnosis in breast cancer screening

**DOI:** 10.1038/sj.bjc.6604824

**Published:** 2009-01-06

**Authors:** S W Duffy, E Lynge, H Jonsson

**Affiliations:** 1 Cancer Research UK Centre for Epidemiology Mathematics and Statistics, Wolfson Institute of Preventive Medicine, Charterhouse Square, London EC1M 6BQ, UK; 2 Institute of Public Health, University of Copenhagen, Øster Farimagsgade 5, Opg. B, DK-1014 Copenhagen K, Denmark; 3 Department of Radiation Sciences, Oncology, Umeå University, Umeå, Sweden

**Sir,**

Your correspondents’ main assertion is that our estimate of lead time in breast screening is too large due to the inclusion of all breast cancer cases in its estimation and that as a consequence our estimate of overdiagnosis is too small. First, we explicitly stated that our excess incidence of 39% was not claimed to estimate overdiagnosis, so they are mistaken on that count. Secondly, we do not find their argument about lead time persuasive, as our estimate of 2.4 years is consistent with sojourn time estimates from studies that used heterogeneity models allowing for some cancers never to progress (Duffy *et al*, 2005; Olsen *et al*, 2006).

Your correspondents’ preferred approach (Zahl *et al*, 2004) of comparing the rise in incidence in a screened age group with the drop in incidence at higher ages is similarly unconvincing because, as was pointed out in the *British Medical Journal* rapid response columns at the time, there was inaccurate specification of screened age groups, insufficient time for the subsequent drop in incidence to be observed and insufficient attention was paid to other factors (notably hormone replacement therapy use).

We are also sceptical about their assertions concerning overdiagnosis in the Malmö Trial. They cite screening in the control group but fail to consider continued voluntary screening in the study group after invitations had ceased.

Whether it is convenient or not for us and for your correspondents, methodological complexities such as lead time are highly influential in the observed incidence during a screening programme. Failure to take these into account leads to implausibly high estimates of the order asserted by your correspondents.
